# Femtosecond laser-assisted intracorneal MyoRing explantation - A novel rescue technique

**DOI:** 10.1016/j.ajoc.2025.102313

**Published:** 2025-03-22

**Authors:** Nikolaus Luft, Stefan Kassumeh, Martin Dirisamer, Siegfried G. Priglinger, Wolfgang J. Mayer

**Affiliations:** Department of Ophthalmology, LMU University Hospital, LMU Munich, Mathildenstrasse 8, Munich, Germany

**Keywords:** Keratoconus, MyoRing, Corneal ring implants, Femtosecond laser, Intrastromal corneal ring segments

## Abstract

**Purpose:**

We describe a case of a keratoconus patient with decreased uncorrected visual acuity after MyoRing implantation, where explantation was indicated.

**Observations:**

A 25-year-old male patient was referred to our institution for evaluation after being deemed unfit for service as a police officer for six months. He had received a MyoRing ring implant in his right eye due to keratoconus. Following the procedure, he experienced decreased uncorrected visual acuity, intolerance to contact lenses, and unsatisfactory visual acuity with glasses. Consequently, a femtosecond laser-assisted removal of the ring implant was performed.

**Conclusions and importance:**

We present a novel femtosecond laser-based “rescue” technique for the removal of continuous corneal ring implants that cannot be extracted via the original intrastromal pocket. The minimal-invasive FS-approach might also be useful for the explantation of other intracorneal implants (e.g. ICRS) or devices that cannot be removed via the original surgical approach.

## 1 Introduction

Keratoconus is the most common corneal ectasia with a prevalence of approximately 1 in 700 individuals.^1^ The main treatment objectives are to maintain visual acuity and halt the progression of corneal ectasia. For visual improvement in advanced cases, rigid gas permeable contact lenses are necessary to neutralize the irregular astigmatism.

In cases of progression, corneal collagen cross-linking is the primary treatment to halt changes in the corneal shape.^2^ In stabilized keratoconus patients who are intolerant to contact lenses, surgical options such as intrastromal corneal ring segments (ICRS) can be considered.^3^

In this case, we present the removal of an intrastromal ring implant (MyoRing) in a 25-year-old patient who complained about decreased uncorrected visual acuity after surgery.

## 2 Case report

A 25-year-old male patient was referred to our institution for evaluation after being unfit for service as a police officer for six months. He had a history of bilateral progressive keratoconus and had undergone mechanical microkeratome-assisted (PocketMaker; Dioptex, Linz, Austria) corneal intrastromal implantation of a 360° polymethylmethacrylate (PMMA) ring implant (MyoRing; Dioptex, Linz, Austria) elsewhere in his right eye six months previously. No other surgical intervention, including corneal cross-linking (CXL), had been performed. His medical records from the last pre-ICR implantation visit elsewhere revealed K-max values (Pentacam HR; Oculus Optikgeräte GmbH, Wetzlar, Germany) of 46.2 Diopters (D) in the right eye and and 47.3 D in the left eye. Uncorrected distance visual acuity (UDVA) was 20/30 in the right eye and 20/40 in the left eye. Spectacle corrected distance visual acuity (CDVA) was 20/20 in both eyes with a manifest refraction of 0.00 -1.00 × 91° in the right eye and −0.25 -0.75 × 66° in the left eye. Moreover, serial corneal tomography scans (Pentacam HR) from the previous three years revealed clinically significant keratoconus progression in both eyes.

**Fig. 1:**
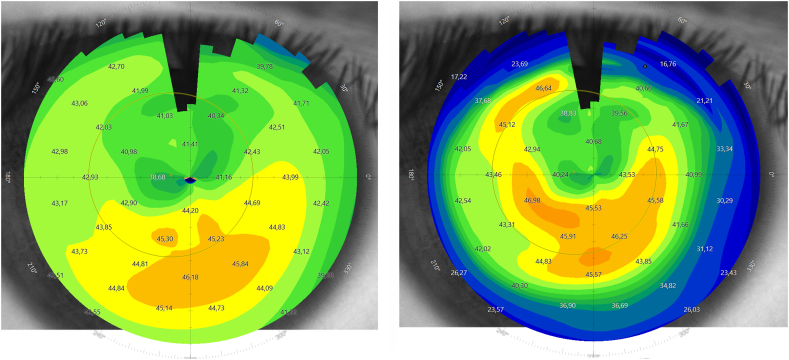
Preoperative corneal topography maps: axial anterior corneal curvature display (left), axial tangential corneal curvature display (right).

**Fig. 2:**
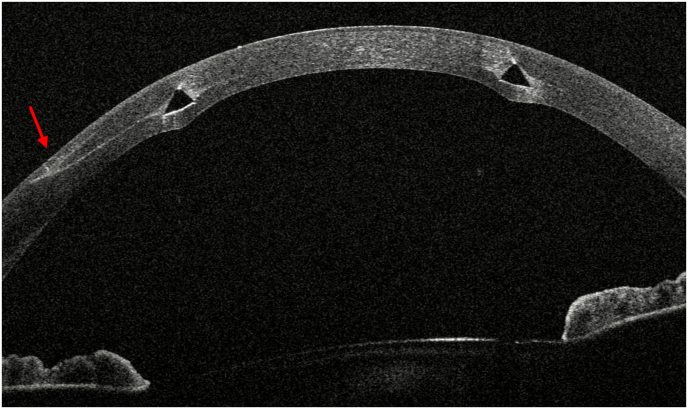
Preoperative horizontal AS-OCT B-scan showing subepithelial stromal hyperreflectivity indicative of stromal fibrosis at the temporal pocket entrance (red arrow).

**Fig. 3:**
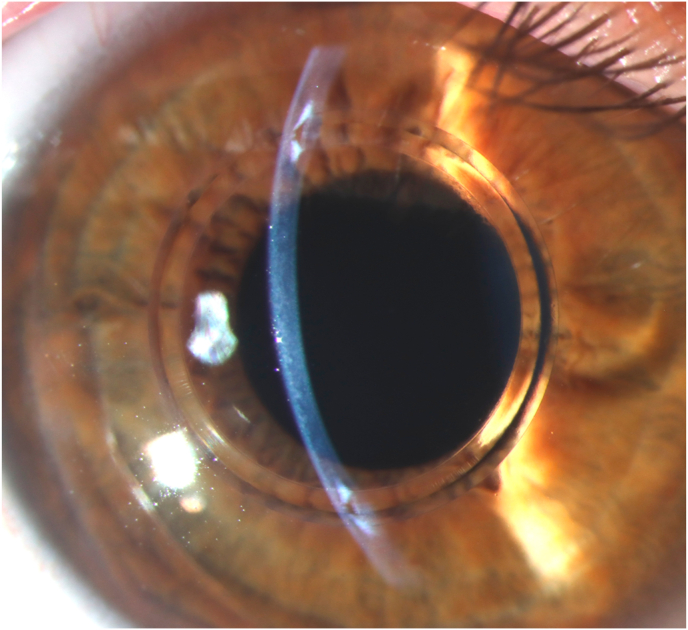
Preoperative slit-lamp photo. The intracorneal ring segment was well-centered. Stromal fibrosis adjacent to the alloplastic PMMA implant was evident.

On initial examination at our institution, UDVA of the right (treated) eye was 20/400 and corrected distance visual acuity (CDVA) with a manifest refraction of +4.50–2.25 D x 14° increased to 20/25. The left (untreated) eye showed an UDVA of 20/50 and a CDVA of 20/25 with a manifest refraction of −0.50 D −1.25 D x 62°. Corneal tomography scans acquired at this initial visit at our institution are displayed in Fig. 1. The ICR was well discernible as a hyporeflective intrastromal structure on anterior segment optical coherence tomography (AS-OCT; MS-39; C.S.O., Florence, Italy; Fig. 2). The ICR was well-centered; however, stromal fibrosis adjacent to the alloplastic PMMA implant was evident on AS-OCT and slit-lamp biomicroscopy (Fig. 3). The patient was contact lens intolerant despite multiple contact lens fitting attempts with rigid gas permeable as well as soft toric contact lenses. Spectacle correction of his refractive error was not tolerated due to debilitating aniseikonia.

With respect to the ICR implanted (right) eye, a conjunct decision was made to remove the implant. From the patient's perspective, implant removal was requested to restore his previous UDVA of 20/30 prior to MyoRing implantation. From a medical perspective, significant stromal fibrosis in vicinity to the implant was already present as early as six months after ICR implantation. Hence, implant removal was advised to prevent further complications such as stromal fibrosis, necrosis, melting or extrusion, all of which are well-known long-term complications of intrastromal PMMA implants like the MyoRing.^4^, ^5^, ^6^, ^7^

Despite microscope-integrated intraoperative AS-OCT guidance (RESCAN700, Carl Zeiss Meditec AG, Germany), a first attempt to explant the ICR implant via the original microkeratome-created stromal pocket failed as the pocket could not be opened due to stromal fibrosis at the temporal pocket entrance (Fig. 2). Consequently, a femtosecond laser-assisted (FS; FEMTO LDV Z8, Ziemer, Switzerland) approach was employed in order to create a novel pocket with a 4.5mm incision at the 90° position (superiorly) through which the ICR could be safely explanted (**Supplementary Video**). The 8.0mm pocket was created at the level of the ICR a depth of 340μm and centered using the femtosecond laser system's onboard AS-OCT guidance system. After FS laser application, the novel pocket was accessed effortlessly using the sharp tip of a small incision lenticule extraction (SMILE) lenticule separator. Thereafter, the blunt spoon-shaped end of the instrument was introduced and - using circular movements - the ICR was freed from peri-implant fibrosis before being grasped and explanting using small tying forceps (**Supplementary Video**). Postoperatively, preservative free ofloxacin eyedrops were prescribed qid for 1 week and dexamethasone 0.1 % eyedrops six times daily were tapered over a course of 6 weeks.

**Fig. 4:**
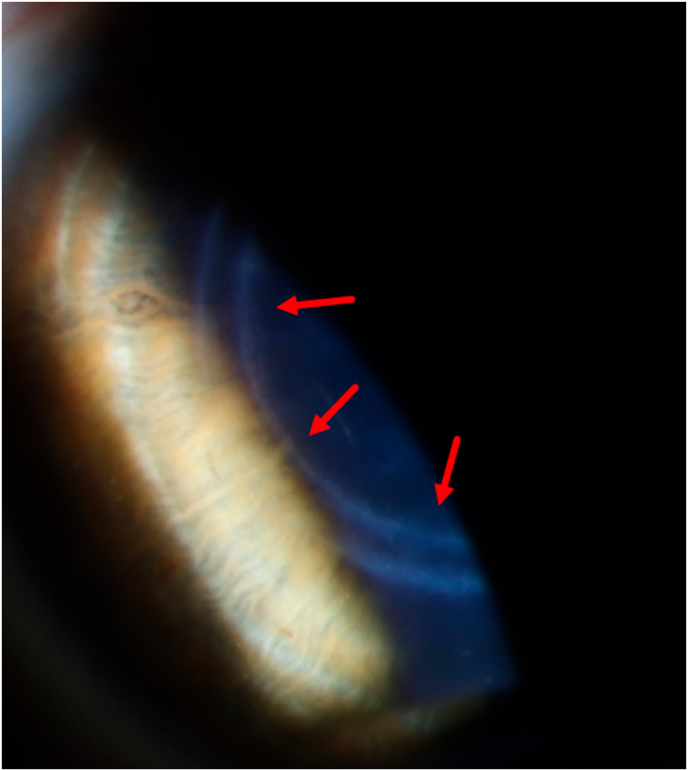
Postoperative slit-lamp photo. Faint circular-shaped stromal fibrosis was visible as indicated by the red arrows.

**Fig. 5:**
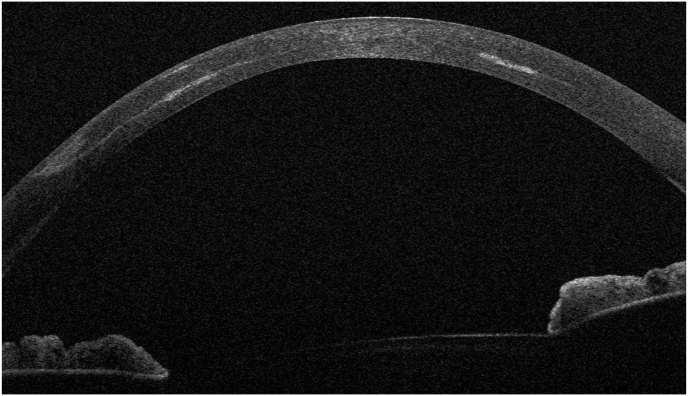
Postoperative horizontal AS-OCT B-scan showing stromal hyperreflectivity indicative of stromal fibrosis at former location of the ICR implant.

**Fig. 6:**
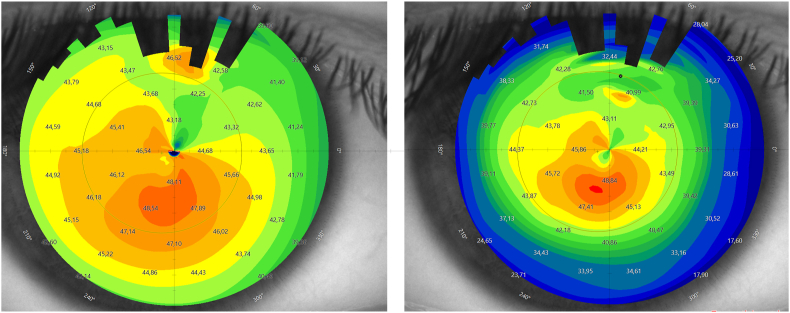
Postoperative corneal topography maps: axial anterior corneal curvature display (left), axial tangential corneal curvature display (right).

Four months postoperatively, faint circular-shaped stromal fibrosis was visible on slit-lamp biomicroscopy (Fig. 4) at the previous location of the ICR and corresponding stromal hyperreflectivity was observable on AS-OCT (Fig. 5). After ICR removal, the patient's UDVA partially recovered to 20/40 and the CDVA decreased to 20/40 with a manifest refraction of 0.00 D −1.75 D x 41°. The post-explantation tomography maps are displayed in Fig. 6.

## 3 Discussion

The MyoRing is a continuous PMMA ring implant with a diameter ranging from 5.00 to 8.00 mm and a thickness ranging from 200 to 320 μm. Unlike intracorneal ring segments (ICRS), which are implanted into corneal stromal tunnels, the MyoRing is implanted into a corneal stromal pocket that can be created with a mechanical microkeratome (PocketMaker; Dioptex, Linz, Austria) or a FS laser.^8^ Both types of implants work by an arc-shortening effect on the ectatic cornea with the aim of flattening the central part and distending the peripheral part of the cornea. ICR and ICRS implants are typically reserved for contact lens intolerant keratoconus patients with a clear central cornea and aim to improve contact lens tolerance and CDVA. In a recent meta-analysis, Struckmeier et al.^9^ found much higher complication rates (e.g., anterior chamber perforations) with ICRS when intrastromal tunnels were created with mechanical dissection as compared with FS laser-assisted techniques. However, data on the safety of the MyoRing, especially with manual pocket creation, is very sparse.^8^, ^9^, ^10^ For instance, Basiony et al.^11^ compared FS laser-assisted MyoRing implantation with manual pocket dissection in terms of safety. In their prospective study of 64 eyes, 21.4 % of eyes with a manual approach experienced implant extrusion, whereas none occurred in the FS laser group.^11^ In a recent study of 118 MyoRing implanted eyes, 14 % required additional surgery such as implant exchange or centration, with 5 % due to refractive overcorrection or hyperopic outcome, as seen in the present case.^12^ The authors noted particularly low predictability of the achievable visual improvement in patients with good preoperative spectacle corrected visual acuity of >20/30, similar to our patient.

In conclusion, we present a novel FS laser-based “rescue” technique for continuous corneal ring implants that cannot be removed via the original intrastromal pocket. This minimally invasive FS-approach may also be useful for the explantation of other intracorneal implants (e.g. ICRS) or devices that cannot be removed via the original surgical approach.

## CRediT authorship contribution statement

**Nikolaus Luft:** Writing – review & editing, Writing – original draft, Supervision, Methodology, Investigation, Conceptualization. **Stefan Kassumeh:** Writing – review & editing, Writing – original draft, Project administration, Methodology, Conceptualization. **Martin Dirisamer:** Writing – review & editing, Writing – original draft, Supervision, Methodology. **Siegfried G. Priglinger:** Writing – review & editing, Writing – original draft, Supervision, Methodology. **Wolfgang J. Mayer:** Writing – review & editing, Writing – original draft, Supervision, Methodology, Investigation, Conceptualization.

## Patient consent

Written consent from the patient was obtained prior to preparing this manuscript.

## Patient consent

Consent to publish this case report has been obtained from the patient in writing.

## Acknowledgements and disclosures

No funding or grant support.

## Authorship

All authors attest that they meet the current ICMJE criteria for authorship.

## Declaration of competing interest

The authors declare that they have no known competing financial interests or personal relationships that could have appeared to influence the work reported in this paper.
